# Sedimentary evolution and controlling factors of Early-Mid Miocene Deltaic systems in the Northern Pearl River Mouth Basin, South China Sea

**DOI:** 10.1038/s41598-021-85369-1

**Published:** 2021-03-17

**Authors:** Yanru Wang, Changsong Lin, Zhongtao Zhang, Bo Zhang, Hanyao Liu

**Affiliations:** 1 School of Ocean Sciences, China University of Geosciences, Beijing, 100083 China; 2 School of Geographical Sciences, Harbin Normal University, Harbin, 150025 China; 3 Shenzhen Branch of CNOOC Ltd., Shenzhen, 518000 China

**Keywords:** Geology, Geophysics, Sedimentology, Seismology

## Abstract

The Pearl River Mouth Basin is located at the northern continental shelf of the South China Sea. Since the early Miocene, the Paleo-pearl river transported a large amount of sediments to the northwest of the basin and resulted in the formation of a large-scale river-delta depositional system, which has become an important oil and gas reservoir in the study area. In the current paper, we investigate the characteristics and evolution of fluvial-deltaic depositional systems and their controlling factors, including sea level change, tectonic subsidence and sediment supply on the basis of 3D seismic, well logging and core data. Early-Mid Miocene succession can be divided into two second-order sequences (CS1–CS2) and eight third-order sequences (S1–S8). Deltaic systems developed in S1–S2 are relatively coarse in grain size, and the delta plain deposits are dominated by thickly stacked (100–180 m) distributary channel sand bodies and interpreted as braided delta depositional system. In the early stage (S1–S2), the braided delta systems mainly distributed in the west of the Baiyun Sag, which were proceeded by a retreat to the south of the Enping Sag along with sharply rising sea level. Following the transgression of S2, the provenance of the Paleo-Pearl River extended to the coastal region of South China, and the papleoclimate changed from warm and humid to dry and cold in the early Miocene, leading to the development of transition of braided river delta to meandering river delta, which was characterized by relatively fine grain deposits. During the deposition of S3–S6, well sorted and rounded fine sandstones of deltaic front deposits accumulated in the study area. The retrogradation to accretion and subsequent progradation of these meander delta systems are attributed to the sea level change in the study area. During the deposition of S7–S8, the delta front retreated to the south of the Enping depression as a result of minor sea level rise, reduction in sediment input, and subsidence rate. This resulted in the development of a wave-controlled deltaic depositional system.

## Introduction

The formation of deltas is affected by multiple factors, including waves, tides and rivers, with the former considered as the most importanta^1,2^. River deltas can be divided into three categories according to the river type and the distance from the source area, namely, fan, braided and meandering river deltas^3^, each having a different control effect on sedimentary sand bodies^4,5^. Braided river deltas are rich in gravel and coarse sands coming from braided rivers controlled by floods^6,7^. Compared with the meandering river delta, the braided river delta is more coarse-grained, with a greater lateral connectivity of the sand bodies and better storage performance. In recent years, an extensive amount of research has been performed on the sedimentary characteristics and depositional patterns of marine braided and meandering river deltas^8–12^, however, studies on the transformation mechanism and the controlling factors of the two kinds of the deltas are not sufficient yet^13,14^.

The Pearl River Mouth Basin is located on the northern shelf margin of the South China Sea. Following the Baiyun tectonic movement in the Late Oligocene^15^, the basin entered a post-rift subsidence stage. In particular, a large amount of calstic sediments from the Paleo-Pearl River were injected, resulting in the development of large-scale delta deposits in the basin. Previous studies^16,17^ have suggested that braided river deltas were dominated in the early Miocene, while meandering river deltas were dominant in the late Early-Mid Miocene. However, the majority of research focuses on the southeast of the Panyu Low Uplift, while work on the delta system of the northern Panyu low uplift and Enping Sag is limited. Furthermore, there is a research gap on the conversion mechanism and evolution process of braided and meandering river deltas, restricting investigations on the delta deposition systems and sedimentary reservoirs in this area. Thus, in the current paper, we explore the sedimentary characteristics and transformation mechanisms of braided and meandering river deltas using 3D seismic, well logging and core data. The migration and evolution processes of deltas in the vertical and planar directions are analyzed in detail, as well as the controlling effects of sea level change, tectonic subsidence and sediment supply on the depositional evolution of different delta types. This paper provides a theoretical foundation for the delta-related prediction of oil and gas reservoirs.

### Study area and geologic setting

**Figure 1:**
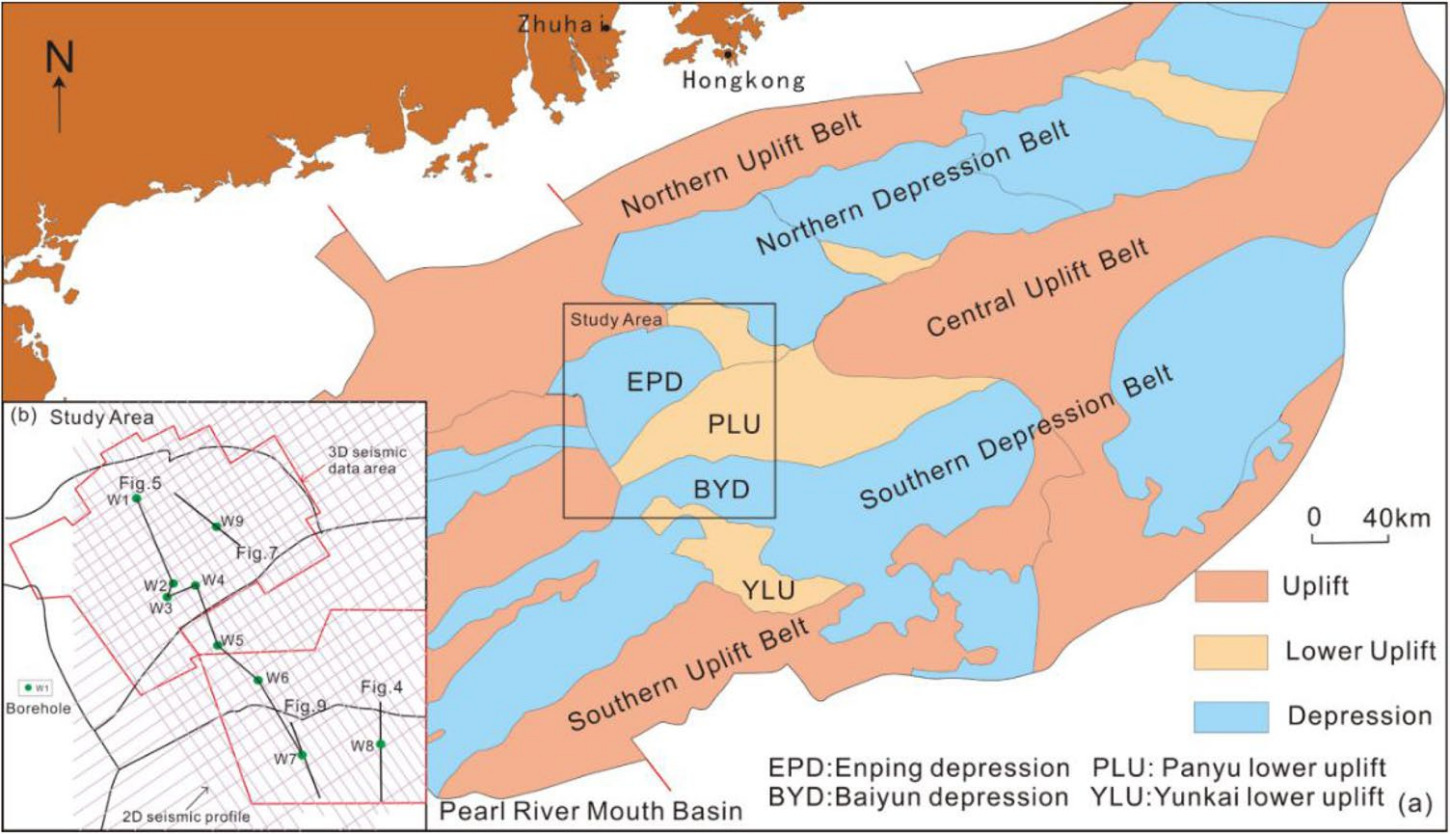
(**a**) Distribution and tectonic unit division of the Pearl River Mouth Basin (modified from Lin et al. 2017), (**b**) location of figures and seismic and well logging data collection points.

The Pearl River Mouth Basin is located in the northern region of the South China Sea, and is described as a Cenozoic extensional basin. It can be divided into five units from north to south: the northern step-fault belt, northern depression belt, central uplift belt, southern depression belt and southern uplift belt^18^ (Fig. 1a). The evolution of the basin can be grouped into three stages^15,19^: (1) the rifting stage during the Eocene-early Oligocene; (2) the post-rifting thermal subsidence stage during the Late Oligocene-Middle Miocene; and (3) the ups and downs of the fault block stage from the Late Miocene to the present. A number of hydrocarbon-bearing structures have been discovered in the northern shallow water shelf area, and the exploration and development of oil and gas reservoirs in the deep-water continental slope area is also a current research focus^19–23^. Therefore, exploring the deposition and evolution laws of the delta from the inner shelf to shelf margin is of great significance for the search for high-quality reservoirs^24^.

**Figure 2:**
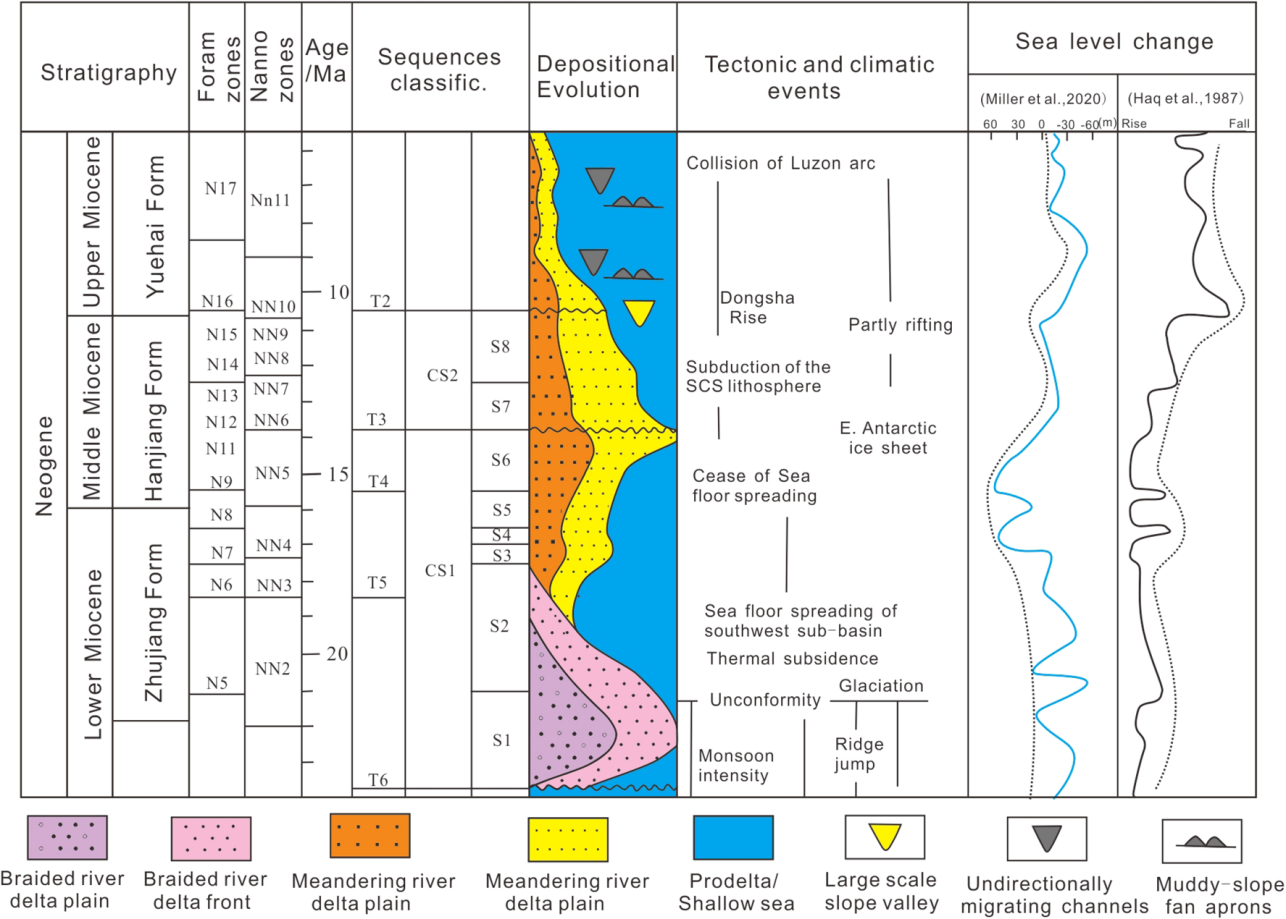
Sequence stratigraphy framework and depositional evolution of the Early-Mid Miocene in the northern Pearl River Mouth Basin (sequence divisions and fossil zones^19,25–27^, tectonic and climatic events^28^).

The study area covers the Enping Sag, the western part of the Panyu Low Uplift and the northwestern part of the Baiyun Sag (Fig. 1b). The Zhujiang Formation and Hanjiang Formation were deposited during the Early-Mid Miocene. The braided river delta with medium-coarse and shoreline sandstones are considered to be deposited in the lower region of the Zhujiang Formation, and in the middle-upper zone of the Zhujiang Formation–Hanjiang Formation, described as a meandering river delta with medium-fine sandstone and a shallow sea shelf with silty mudstone and mudstone, respectively (Fig. 2).

### Data and research methods

This study is based on the comprehensive analysis of high resolution 3D seismic data (provided by the China National Offshore Oil Corporation, Shenzhen) and nine well logs and cores. The well logs, which were revised by the cores, were employed to recognized the lithofacies and sedimentary facies, as well as to distinguish the braided and meandering river deltas. The 3D seismic profiles were revised by well VSP (vertical seismic profile) data in order to identify the seismic facies and trace seismic unconformities. The root-mean-square amplitude profiles aided in depicting the distribution of various sedimentary facies in different depositional systems.

## Results

### Lithofacies associations and depositional environments

Based on the analysis of core data and well logs in the study area, a series of delta sedimentary systems can be identified in the Early-Mid Miocene sequence. The main lithofacies associations are interpreted as the products of the depositional environments as follows (FA1-FA13): (1) delta plain deposits; (2) delta front deposits; (3) predelta deposits; and (4) shallow shelf deposits.

### Lithofacies associations FA1–FA2: braided river delta plain deposits

**Figure 3:**
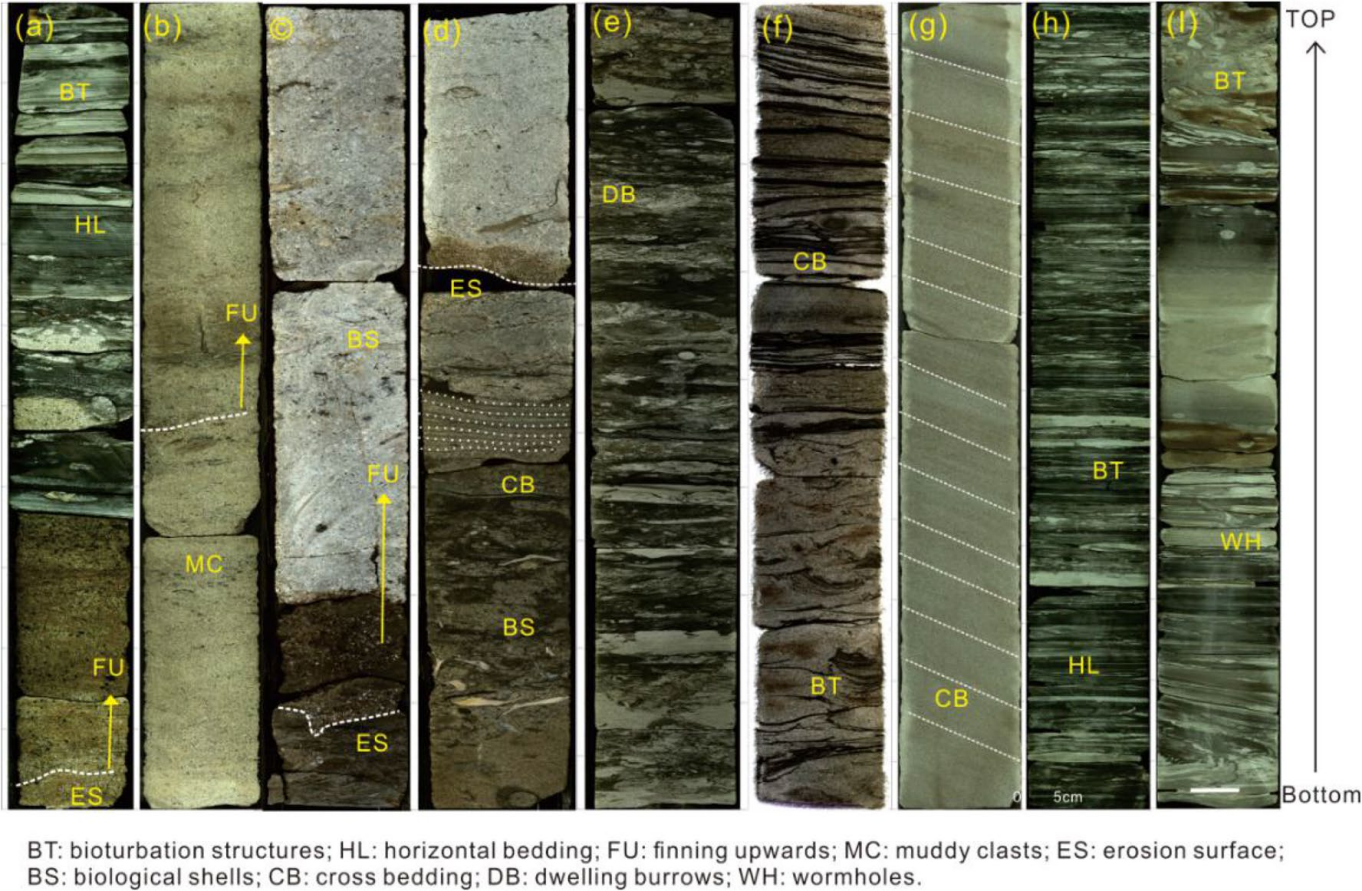
Core photos of braided river delta and meandering river delta deposits: (**a**) Braided interdistributary bay deposit(FA2); (**b**) Braided distributary channel deposits(FA1) (location of cores **a**, **b** shown in Fig. 5, borehole W3 in Fig. 1b, 2620–2627 m depth; S1); (**c**) Subaqueous distributary channel deposits of braided river delta front: (FA3); (**d**) Estuary dam deposits of braided river delta front(FA4) (location of cores **c**, **d** shown in Fig. 5, borehole W4 in Fig. 1b, 2164–2180 m depth; S2); (**e**) Distal bar deposits of braided river delta front(FA5); (**f**) subaqueous interdistributary bay deposits of braided river delta front(FA6) (location of cores **e**, **f** shown in Fig. 5, borehole W2 in Fig. 1b, 2297–2289 m depth; S2); (**g**) distributary channel deposits of meandering river delta plain(FA7); (h) Distal bar deposits of meandering river delta plain (FA11); (**i**) subaqueous interdistributary bay deposits of meandering river delta plain (FA12) (location of cores g-i shown in Fig. 5, borehole W3 in Fig. 1b, 2044–2039 m depth; S3).

These lithofacies associations are composed of FA1 and FA2 (Table 1). The principle component of FA1 is yellow-white massive pebbly sandstone, which is poorly sorted and presents obvious erosion surfaces in the interior. The sandstone bodies are approximately 30–60 m in thickness, and display a general fining-upward pattern (Fig. 3a). FA2 is composed of thin grayish black mudstone, with sandstone lenses or sandstone interbedded at the bottom and abundant bioturbation structures, and small scale horizontal or wavy cross bedding (Fig. 3b).

**Table 1:** Main lithofacies associations of braided and meandering river deltas from the Early-Mid Miocene in the study area.

Lithofacies associations	Depositional characteristics	Depositional environments	Depositional facies
FA1	Thick beds of pebbly coarse and medium sandstone, poorly sorted, graded and massive bedding, with obvious scouring surface and dark mud boulder, partly intercalated with muddy laminae; several isolated wormholes; finning-upward facies succession; fir-tree or box-shaped log curve	Braided distributary channel	Braided river delta plain
FA2	Thin layer of dark mudstone with large sandstone lenses at the bottom, gradually changes upwards to fine sandstone with a large number of argillaceous layers; small scale horizontal bedding and wavy bedding; top layer of bioturbation structures; low-amplitude dentation log curve	Braided interdistributary bay	
FA3	Medium to thick beds of pebbly medium sandstone, fine sandstone, poorly sorted, occasional biological shells, massive bedding; box-shaped log curve	Subaqueous distributary channels	Braided river delta front
FA4	Medium to thin beds of medium sandstone, calcareous fine sandstone, poorly sorted, massive bedding and small scale trough cross bedding; internal scouring surface, occasional burrows and biological debris; funnel-shaped log curve	Estuary dam	
FA5	Medium to thin beds of argillaceous siltstone; abundant biodisturbed structures; few isolated large wormholes; small scale bimodal cross bedding; medium-amplitude funnel-shaped log curve	Distal bar	
FA6	Thin and silty mudstone on the top, interbedded sand-mud or sand ball-bearing; biodisturbed structures and wormholes, small scale wavy bedding; low-amplitude dentationlog curve	Subaqueous interdistributary bay	
FA7	Thick beds of sandstone, fine sandstone, massive bedding and small scale cross bedding; well sorted and rounded; bell- or box-shaped log curve	Distributary channel	Meandering river delta plain
FA8	Thin siltstone, silty mudstone or mudstone, wavy bedding, biodisturbed structures, low-amplitude linear trend log curve	Interdistributary bay	
FA9	Medium to thin beds of fine sandstone, well sorted; abundant bioturbation structures and biological burrows in thin beds of calcareous siltstone at the top; occasional bioclastics, large scale tabular cross bedding; bell-shaped or box-shaped log curve	Subaqueous distributary channels	Meandering river delta front
FA10	Medium to thin beds of fine sandstone, intercalated pebbly sandstone and siltstone, well sorted and rounded, small scale tabular and wedge-shaped cross bedding; funnel shaped log curve	Estuary dam	
FA11	Thin beds of interbedded fine sandstone and argillaceous siltstone, burrows and wormholes in the siltstone; medium funnel shaped log curve	Distal bar	
FA12	Medium to thick beds of interbedded silty mudstone, mudstone, argillaceous siltstone, large number of biodisturbed structures, horizontal bedding; low-amplitude dentation log curve	Subaqueous interdistributary bay	
FA13	Thick bed of mudstone, silty mudstone, intercalated thin siltstone, horizontal bedding, biodisturbed structures, low-amplitude dentation log curve	Predelta mud/shelf mud	Predelta /shallow shelf

These lithofacies associations (FA1–FA2) are interpreted as braided river delta plain deposits, including braided distributary channels (FA1) and braided interdistributary bay deposits (FA2). The thick thinning upward pebbly sandstone (FA1) represents the braided distributary channel deposits, which is easily recognized on the well logs by a clear funnel or boxed shaped gamma ray (GR) pattern (Fig. 5). The thin mudstone with horizontal bedding in the FA2 lithofacies associations is interpreted as braided interdistributary bay deposits, with a serrate GR well-log profile. The sand factor of the braided river delta plain is relatively high, ranging between 77 and 90% and generally originated from the braided distributary channel.

### Lithofacies associations FA3–FA6: braided river delta front deposits

These lithofacies associations are composed of FA3–FA6 (Table 1). FA3 is composed of medium and thick beds of variegated medium to fine grained pebbly sandstone, which is poorly sorted and subangular, with large scale massive and cross bedding and irregular scoured surfaces. Biological shells can be seen at the bottom of white sandstone (Fig. 3c). FA4 is composed of medium and thin beds of variegated medium sandstone and calcareous fine sandstone with massive bedding or small scale trough cross-bedding, with a general upward coarsening. Wormholes and biological shells can be seen in the lower siltstone (Fig. 3d). FA5 is composed of gray argillaceous siltstone or silty mudstone with small scale horizontal or tabular cross-bedding. The argillaceous is usually located at the top and the lower siltstone contains a large number of biological disturbance structures (Fig. 3e). FA6 is an interbedded layer of thin gray and silty mudstone with wavy cross bedding. The bioturbation structure is extremely developed, with occasional isolated large wormholes (Fig. 3f).

The lithofacies associations of FA3–FA6 are interpreted as braided delta front deposits due to their upward thickening. The fine pebbly sandstone (FA3) at the top is interpreted as subaqueous distributary channel deposits, with box- or bell-shaped GR well logs. The proximal delta front deposits contain thin beds of fine sandstone and siltstone (FA4), and can been interpreted as an estuary dam with funnel shaped GR logs. The argillaceous siltstone with horizontal bedding (FA5) is the distal delta front the deposits and can been interpreted as distal bar deposits. The bimodal cross bedding in the pelitic strip may be related to the scouring and backflow of the tides^26,29,30^. The dark mudstone with an abundant biodisturbed structure of FA6 represents the subaqueous tributary bay deposits. The sand percentage of the braided river delta front ranges between 42 and 60%, and the distal drops to approximately 33%, which is slightly lower than that of the braided river delta plain.

### Lithofacies associations FA7–FA8: Meandering river delta plain deposits

FA7 is generally composed of thick gravel-bearing coarse-medium sandstone that is well sorted and rounded, with an extensive structure, medium and small scale cross bedding and a scouring-filling structure (Table 1). The single sand body thickness is approximately 30–50 m (Fig. 5). FA8 is composed of medium and thin bed mudstone and silty mudstone, with small scale wavy cross bedding and biological disturbance structures and burrows abundant in the cores.

The lithofacies associations of FA7–FA8 are interpreted as meandering river delta plain deposits, and include distributary channel (FA7) and distributary bay (FA7) deposits (Table 1). The upwardly thinning medium-thick layer of the gravel-bearing coarse sandstone (FA7) represents distributary channel deposition, and the GR curve is bell- or box-shaped. Middle-thin mudstones (FA8) are interpreted as distributary bay deposits, with linear shaped GR well logs (Fig. 5). The sand content of the meandering river delta plain is relatively high, reaching levels greater than 90%, and it is located in northern Enping Sag.

### Lithofacies associations FA9–FA12: Meandering river delta front deposits

FA9 is composed of a medium-thin layer of fine sandstone with large scale tabular cross-, block-, and graded-bedding, and a sand body thickness generally within 0.5–5 m. The top region includes a thin layer of calcareous siltstone, which contains biological shells and a large number of biological burrows. The erosion surface is generally developed at the bottom and is in abrupt contact with the underlying mudstone (Fig. 3g). The lithofacies associations of FA10 exhibit an upwardly thicker anti-rhythmic structure. The upper part contains fine sandstone with thin layers of gravel-bearing sandstone, while the lower part compromises siltstone or argillaceous siltstone, with small scale cross bedding. FA11 is composed of a thin layer of interbedded fine sandstone and argillaceous siltstone, with wavy cross and flaser bedding, and local collapse and deformation structures. There are abundant burrows and wormholes in the siltstone (Fig. 3h). FA12 is composed of thin layers of silty mudstone and mudstone with horizontal bedding and a large amount of bioturbation structure and wormholes (Fig. 3i).

The lithofacies associations of FA9–FA12 are interpreted as meandering river deltas of frontal deposits. FA9 represents subaqueous distributary channel deposits with bell- or box-shaped GR well logs. Multiple channels are often continuously stacked vertically. The thin layer of upwardly thickening siltstone-fine sandstone (FA10) represents estuary bar deposits with funnel shaped GR well logs and is often vertically superimposed with the subaqueous distributary channel. Siltstone is increasingly observed towards the distal of the delta front, and is frequently interbeded with fine sandstone. The lithofacies associations of FA11 may represent the distal bay deposits, while the deformation structure at the bottom of the core may indicate slope deposits (Fig. 3H). The thin mudstone of FA11 indicates a relatively lentic environment which may represent subaqueous tributary bay deposits. It facilitates the survival of organisms, and is thus abundant in biological disturbance structures and wormholes. The sand factor of the meandering river delta front ranges from 15 to 40%, which is lower than that of the braided river delta front.

### Predelta-shallow shelf deposits

FA13 is composed of thick and dark gray mudstone and silty mudstone, intercalated with thin fine sandstone or siltstone, exhibiting a well-developed horizontal bedding and a large amount of biological disturbance structures. FA13 is interpreted as a predelta-shallow shelf deposit, with a low-amplitude linear trend in the GR log. It is located at the bottom of the delta front, representing the deep-water deposit on the slope.

### Seismic facies

Seismic facies refers to a 3D seismic unit composed of seismic reflections that can be determined within a certain area^31^. It is characterized by the parameters of internal reflection structure^32^, continuity, amplitude, frequency etc., and reflects the lithological combinations and sedimentary characteristics of sedimentary rocks^33^. The drilling and 3D seismic data of the study area are relatively complete and of good quality. Based on the comprehensive analysis of 3D seismic profiles and well logs, two types of seismic facies have been identified as representative delta plain-delta front deposits (Table 2).

**Table 2:** Major seismic facies characteristics of deltaic systems deposits during the Early-Mid Miocene.

Seismic facies	Seismic facies characteristics					Depositional interpretation	Samples
	Internal reflection patterns	Continuity	Amplitude	Frequency	External form		
**SF1**							
SF1-1	Sub-parallel to messy	Medium-poor	Medium–high	Medium	Sheet	Braided river delta plain	
SF1-2	Sub-parallel to parallel	Medium-strong	Medium–high	Medium	Sheet	Meandering river delta plain	
**SF2**							
SF2-1	Oblique progradation	Medium-poor	Medium	Medium–low	Sheet	Delta front	
SF2-2	Imbricate progradation	Medium	Medium	Medium	Sheet	Delta front	
SF2-3	Tangent-oblique rogradation	Medium-poor	Medium–high	Medium	Sheet/lens	Delta front/slope	
SF2-4	S-shaped-oblique progradation	Medium	High	Medium–high	Sheet/lens	Delta front/slope	

Seismic faces 1 is a sub-parallel to messy/parallel seismic reflection pattern with medium to poor continuity, medium to strong amplitude, medium frequency, and a sheet-like external form. The wells drilling of the seismic facies indicate that seismic facies 1 represents delta plain deposits. The seismic reflection event with the strongest amplitude represents distributary channel deposits, and weaker amplitude represents interdistributary bay deposits. The seismic faces of messy seismic reflection and poor continuity (SF1-1) indicate the braided river delta plain. The distributary channel is unstable due to the influence of seasonal floods, while the seismic reflection continuity of the meandering river delta plain is relatively high (SF1-2).

Seismic facies 2 mainly corresponds to the progradational seismic facies in the study area, and can be grouped into four components according to the progradational reflection configuration. SF2-1 is an oblique progradation reflection, with a sheet-shaped medium to poor continuity, middle amplitude and mid-low frequency seismic reflection. SF2-2 is an imbricate progradation reflection, with a sheet-shaped medium continuity, and mid-frequency and mid-amplitude continuous seismic reflection. The wells penetrating the seismic facies demonstrate that SF2-1 and SF2-2 represent the estuary dam or subaqueous distributary channel deposits of the delta front. The thickness of the progradation is thin and is common in the meandering river delta front, with a core distribution in the Enping Sag and northern Panyu Low Uplift. SF2-3 compromises tangent-oblique progradation reflection, with sheet/lens-like medium to poor continuity, mid-frequency and medium to strong amplitude seismic reflection. SF2-4 represents S-shaped-oblique progradation reflection, with sheet/lens-like medium continuity, a strong frequency and medium to strong amplitude seismic reflection. The wells penetrating the seismic facies demonstrate that both SF2-3 and SF2-4 represent delta front/slope deposits, such as the subaqueous distributary channels of the braided river delta front or the proximal estuary dams of the meandering river delta front. The thickness of the progradation is larger than that of SF2-1 and SF2-2, indicating a deeper water environment and sufficient provenance during the sea level decline.

### Sequence and depositional evolution

Sequence refers to the “stratigraphic unit composed of a set of genetically related and relatively integrated strata, with the top and bottom bounded by the unconformity or the corresponding conformity”^34,35^. Characteristical features include truncation, onlap and downlap^36–38^. Despite the extensive research on the classification of the stratigraphic sequence of the Early-Mid Miocene in the study area^39^, a consensus remains to be agreed upon. A composite sequence^27^, the definition adopted in this paper, emphasizes the sedimentary cycle controlled by the regional base level and is limited by the regional unconformity^27,40^. Composite sequences are core to the establishment of a regional sequence stratigraphic framework^27,41^, and the secondary sequence boundary can be further identified in its interior, with the drilling data employed for the subsequent correction. The age of the sequence boundary is determined via calcareous ultramicrofossil and foraminifer fossils^18,25,42^.

Based on the analysis of 3D high-resolution seismic and well logging data, two composite sequences (CS1–CS2) were identified in the study area during the Early-Middle Miocene (Fig. 2), corresponding to two large regional cycles. According to the biostratigraphic calibration, the duration of the composite sequence is approximately 3.3–10 Ma, and thus can be roughly regarded as a secondary sequence^43^. Composite sequences can be further divided into eight sequences (S1–S8) (Fig. 2), with a sequence duration of approximately 0.5–3.5 Ma, implying a third-order sequence^43^.

### Sequence structure and systems tract

CS1 roughly corresponds to the bottom of the Zhujiang Formation and Hanjiang Formation, with a time span of approximately 23.8–13.8 Ma. The lower CS1 zone (T6 reflection interface) is of regional unconformity, indicating the obvious truncation unconformity in the southern Panyu Low Uplift and the northern slope of the Baiyun Sag. Furthermore, this zone is made up of the fluvial deposits of the Zhuhai Formation, while the top CSI region compromises the braided river delta deposits of the Zhujiang Formation. Seven weak truncation or onlap unconformities can be identified in CS1, dividing it into six sequences (S1–S6). The thickness peaks at S2 and gradually thins upwards, while the thicknesses of S1 and S4–S6 are approximately equal. Each sequence can be divided into Lowstand, Transgressive, Highstand and Falling-stage systems tract according to the secondary transgression and recession. The Lowstand systems tract is characterized by tangent-oblique progradation reflections and progressive deltas, which can only be identified in S2 of the Baiyun Sag. The Transgressive systems tract is composed of mudstone and thin bedded sandstone deposits, with moderate-weak amplitude and moderately continuous reflection characteristics on the seismic section. The Highstand systems tract is characterized by various progradation reflection on the seismic section. The falling-stage systems tract can only be identified in S6 on the western region of the Baiyun Sag, which is characterized by the S-shaped-oblique progradation reflection, suggesting delta front deposits.

CS2 corresponds to the middle and upper components of the Hanjiang Formation, with a time span of approximately 13.8–10.5 Ma. The lower CSB2 zone (T3 reflection interface) appears as an obvious truncation unconformity on the seismic profile of the Baiyunxi Sag, and gradually transitions to a parallel unconformity in the northern shelf. Three weak truncation unconformity-conformities can be identified in CS2, which is further divided into two sequences (S7–S8). The S7 and S8 thicknesses are almost equal and slightly thicker than those of S4–S6. The sequence is composed of Lowstand, Transgressive, and Highstand systems tract. The Lowstand systems tract is located in the western region of the Baiyun Sag, which is characterized by thick wedge-shaped progradation seismic reflection. Conversely, the eastern Baiyun Sag does not present signs of the Lowstand systems tract. The Transgressive systems tract is characterized by medium-weak amplitude and medium continuous parallel reflections. Thin sandstones in the northern shelf are represented of (subaqueous) distributary channels, and thick mudstones are distributed in the southern shelf marginal area. The Highstand systems tract is characterized by the low-angle oblique progradation reflections, of which the high amplitude represents the subaqueous distributary channel deposits, and the medium-weak amplitude represents the slope argillaceous deposits.

### Depositional system and depositional evolution

The braided and meandering river delta deposits in the study area were formed during the Early-Mid Miocene. The spatial distribution of the delta types in the sequence framework reflects the sedimentary evolution of the delta depositional system^44–46^.

### Braided river delta depositional system

**Figure 4:**
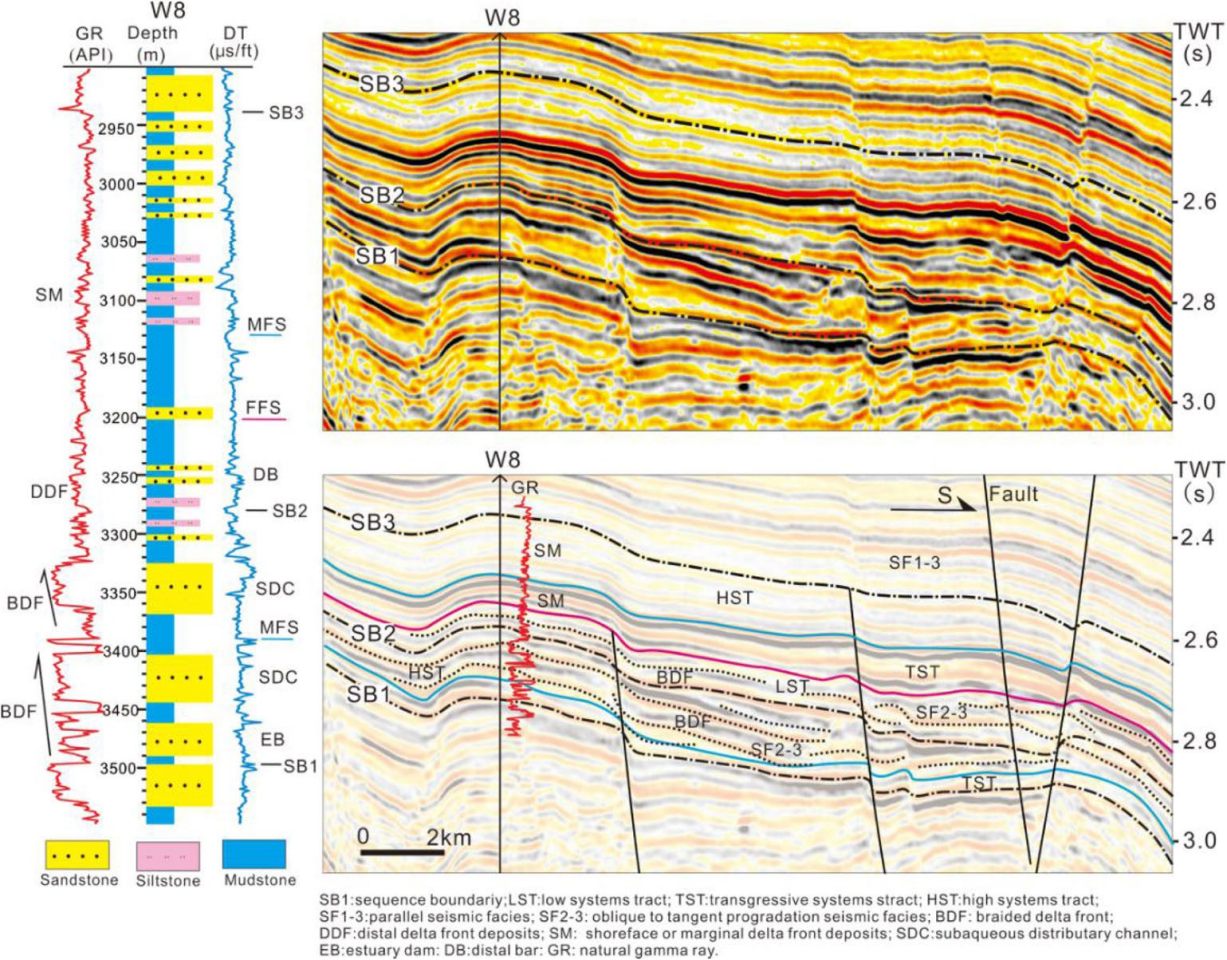
An interpreted seismic profile calibrated to borehole W8 showing the deposits of the braided river delta front at S1 in the northwest Baiyun Sag (see Fig. 1b for location of the profile). The deltaic deposits have characteristic thick bedded foresets with an upward-coarsening grain-size (borehole W8 log curve).

**Figure 5:**
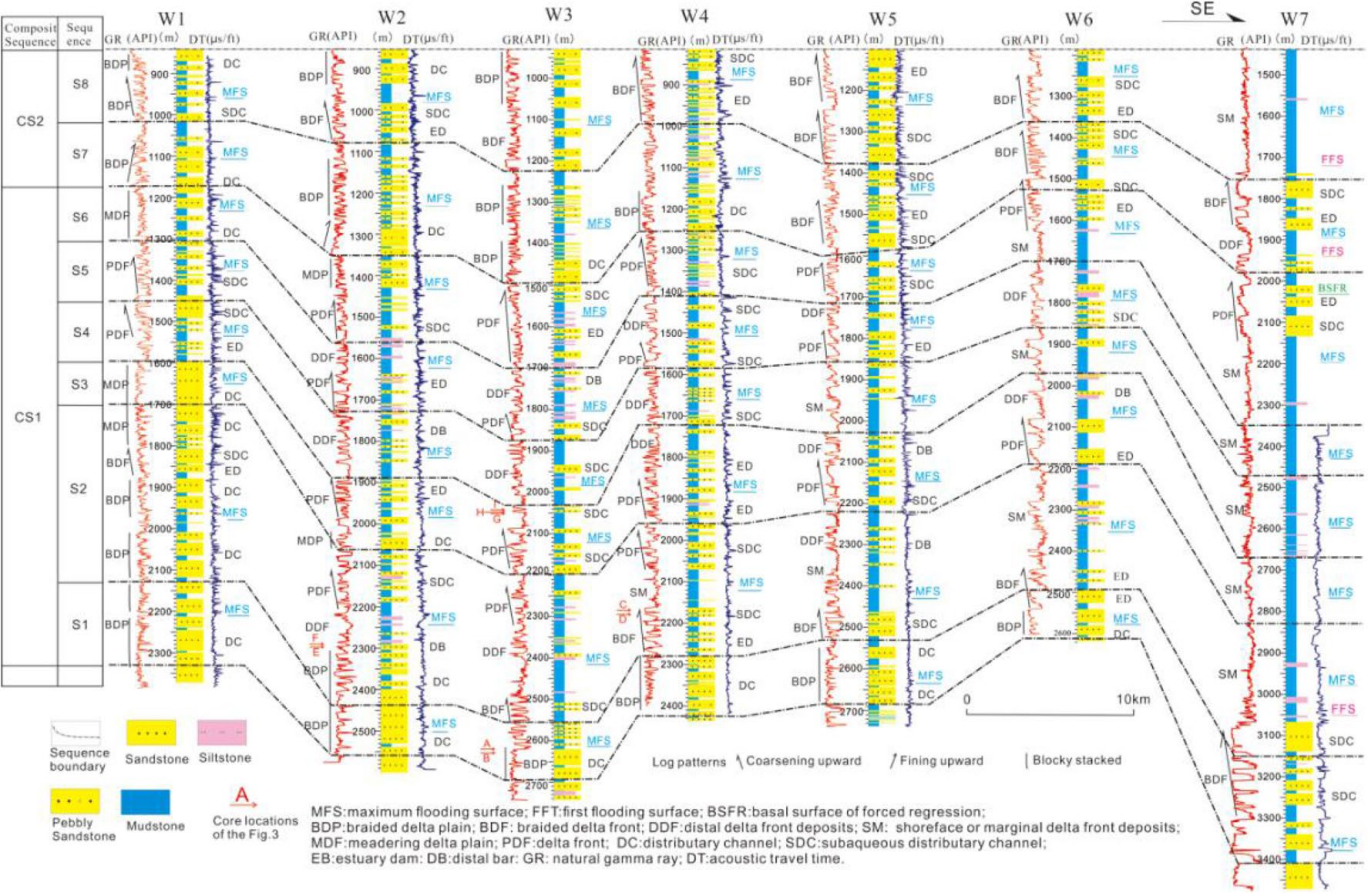
Well log correlation of depositional facies of sequences S1–S8. Note the transition from the braided river delta deposits at the bottom to the meandering river delta deposits and the transition from the delta plain deposits in the northwest to the pre-delta/slope deposits in the southeast at the uppermost region of the study area (location of the log correlation shown in Fig. 1b). The braided river deltiac systems (S1–S2) are characterized by thick bedded of pebbly coarse grained distributary channel deposits in the delta plain and subaqueous distributary channel deposits in the delta front. The meandering river delta systems (S3–S8) exhibit thin or no delta plain deposits and a middle-thick bed of middle-fine sandstone in the estuary dam deposits in the delta front. The location of the cores are shown in the W2, W3, W4 and W5 boreholes.

**Figure 6:**
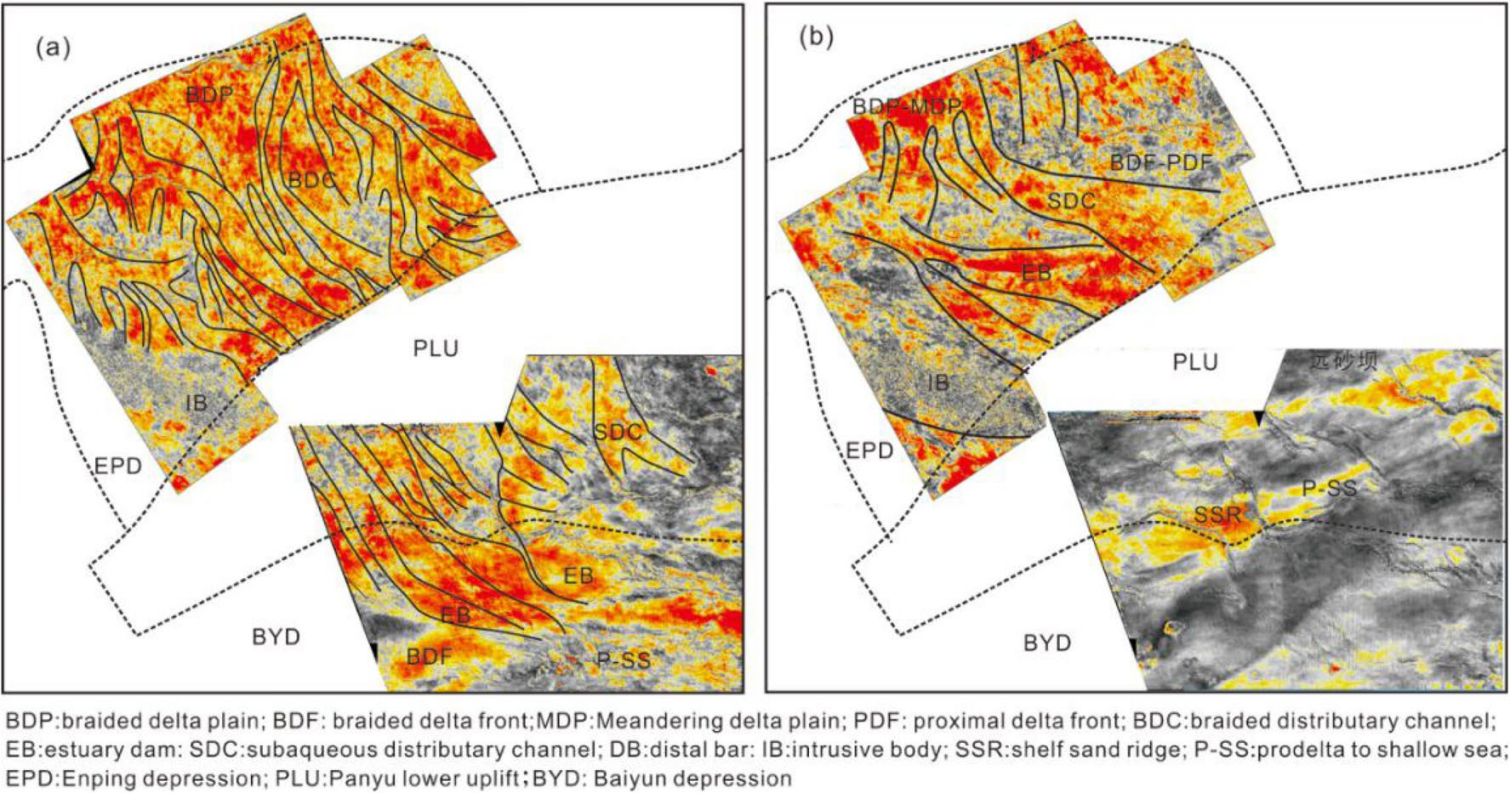
Depositional interpretation of the root mean square amplitude slice of the Braided river delta systems in the Highstand systems tract of S1 (**a**) and transitional characteristics of the Braided river delta and Meandering river delta systems in the Highstand systems tract of S2 (**b**). The Braided river delta systems are characterized by large scale straight distributary channels in the delta plain, whereas the transitional delta systems exhibit small scale or no delta plain deposits and several curved subaqueous distributary channels in the delta front.

The thick wedge-shaped progradation seismic reflections and box or funnel-shaped thick sandstone on the well logs clearly indicate the braided river delta (Figs. 4 and 5). This can be observed in the Transgressive and Highstand systems tract of S1 and the Lowstand and Transgressive systems tract of S2. The braided river delta plain is dominated by braided channel sandstone, and it is located in the Enping Sag (Fig. 6a). The braided river delta front is composed of subaqueous distributary channels and estuary bar deposits, and is located between the southern region of the Panyu Low Uplift and the northern zone of the Baiyunxi Sag (Figs. 5 and 6a). The sand body in the west is obviously thicker than that of the east, indicating the role of the former as the principle provenance channel.

The transgression reached its maximum during the Transgression systems tract of S2, while the braided delta front retreated to the Enping Sag, and the Panyu Low Uplift evolved into a predelta of shallow shelf deposits, covering a thick layer of shelf mudstone (Figs. 5 and 6b). The fine sandstone exhibits a thickness of 5–10 m, is interbeded with mudstone and has box- or funnel-shaped GR logs (Fig. 5). This represents the superposition of multi-period subaqueous distributary channels and estuary dam deposits in the vertical direction. In the Highland systems tract of S2, the root mean square amplitude slice (Fig. 6b) reveals that the distributary channels is concentrated within the center of Enping Sag, with curvature channels greater than those of S1. This suggests the transition from the braided river delta to the meandering river delta and may be attributed to the sharp rise in the sea level and weak provenance supply. The sedimentation rate is less than the growth rate of the accommodated space, resulting in the finer grain size of the sediment.

### Meandering river delta depositional system

**Figure 7:**
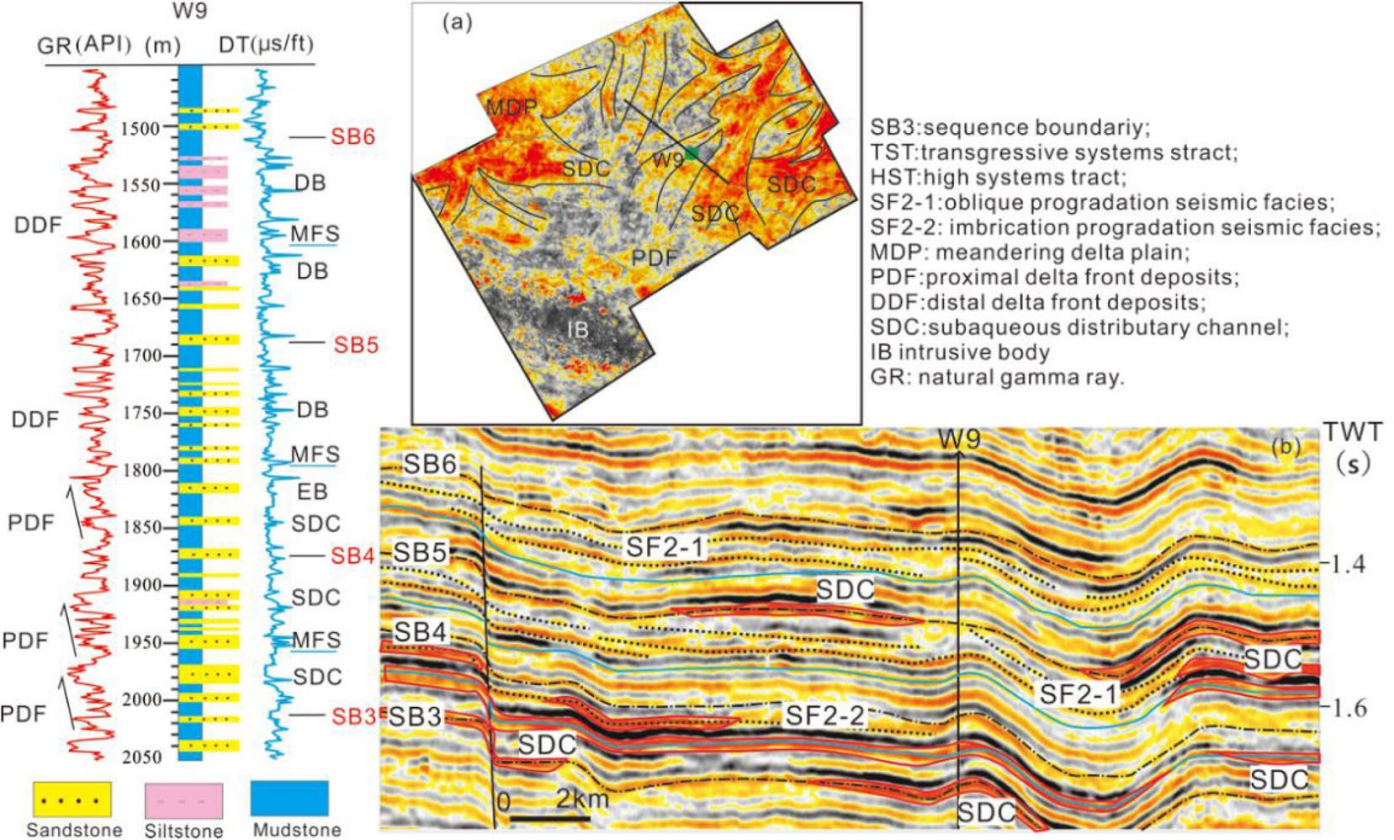
3D seismic root mean square amplitude slice of the Highstand systems tract of meandering river delta systems in S4 (**a**) and an interpreted seismic profile calibrated to borehole W9 showing the deposits of the meandering river delta front from S3 to S5 in the Enping Sag (**b**). The subaqueous distributary channels were divided into three branches on the plane and superimposed vertically on the seismic profile, with a thickness of 3–5 m.

**Figure 8:**
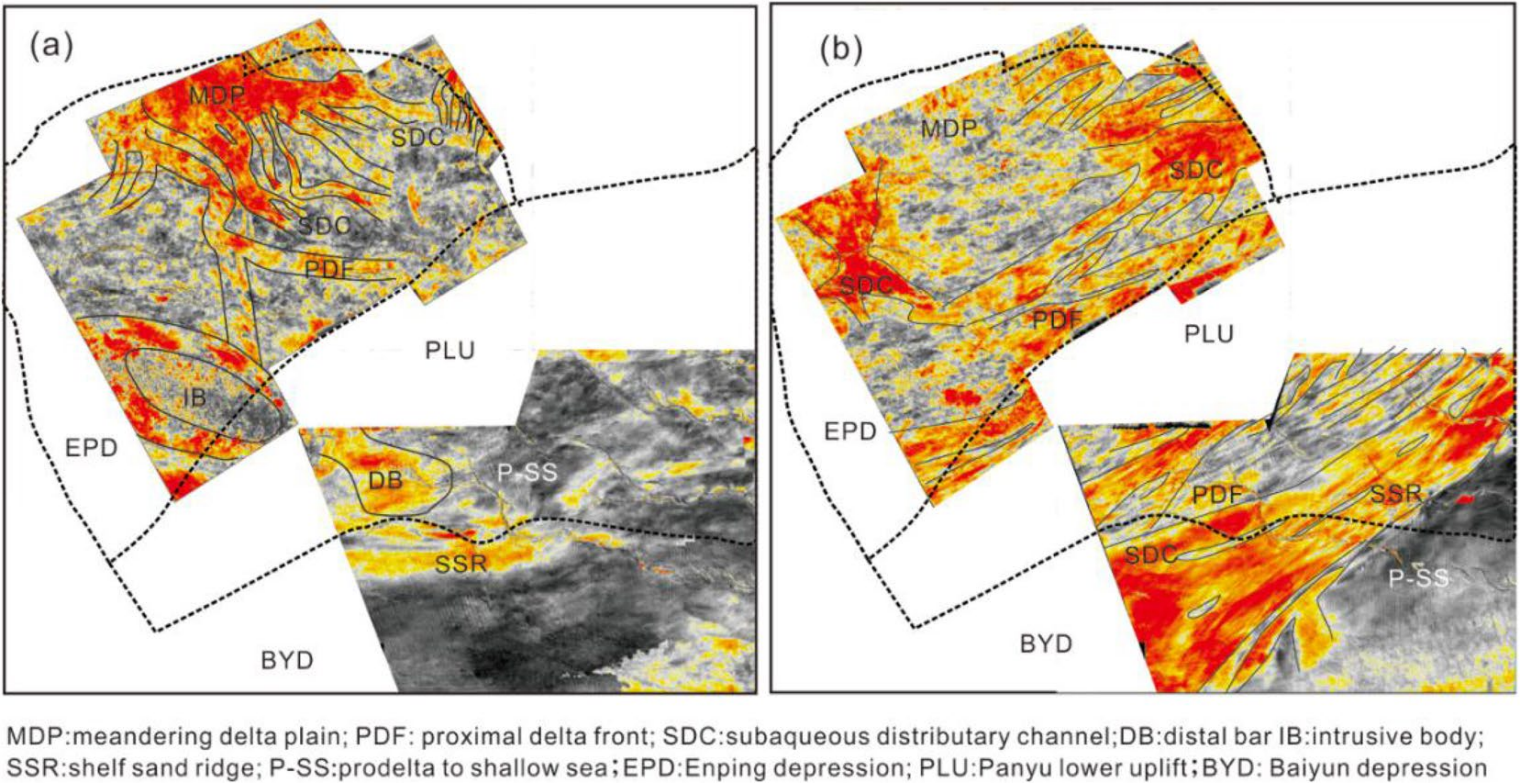
Depositional interpretation of the root mean square amplitude slice of the river-controlled meandering delta deposits in the Highstand systems tract in S5 (**a**) and wave-controlled meandering river delta deposits in the Highstand systems tract in S7 (**b**). The river-controlled delta was bird-foot shaped and the subaqueous distributary channels exhibited a bigger curvature than that of the wave-controlled delta, which was lobate-shaped.

The cores and well logging data reveal that the meandering river delta was developed during the deposition of S3–S8 in the study area. During the deposition of S3–S5, the meandering river delta front was located in the Enping Sag, and then advanced to the Baiyun Sag in S6, where it subsequently regressed to the Panyu Low Uplift during S7–S8. The evolution of the meandering river delta within the sequence stratigraphic framework varied with period. During the deposition of S3, the subaqueous distributary channel of the meandering river delta front developed abnormally (Fig. 7b) and the sand bodies from different periods were superimposed vertically in well logging profile (Fig. 5). The root-mean-square amplitude slice indicates the movement of the channels towards the SE, suggesting possible origin of the clastics to be the northwest of the study area. During the deposition of S4, the subaqueous distributary channel of the meandering river delta front was divided into three branches in the Enping Sag; SE, SSW and SW respectively, with the SE and SW observed as the dominant directions (Fig. 7a). Furthermore, the clastics generally originated from the NW and NE in S4. At the same time, the predelta to shallow shelf deposits were developed in the Panyu Low Uplift and Baiyun Sag (Fig. 5). During the deposition of S5, the meandering river delta plain advanced to the northern zone of the Enping Sag, and the delta front extended to the Panyu low uplift. The subaqueous distributary channel narrowed and the curvature increased. The flow direction was changed to the SE dominant and SW subordinate directions (Fig. 8a). The delta front consistently lingers in the northern zone of the Panyu Low Uplift during the evolution of the meandering river delta in S3–S5, and the channel is obvious and bird-foot in the root–mean–square amplitude slice (Figs. 7a and 8a). This indicates the minimal impact of the global changes in the sea level on the delta, with the river having a greater influence.

**Figure 9:**
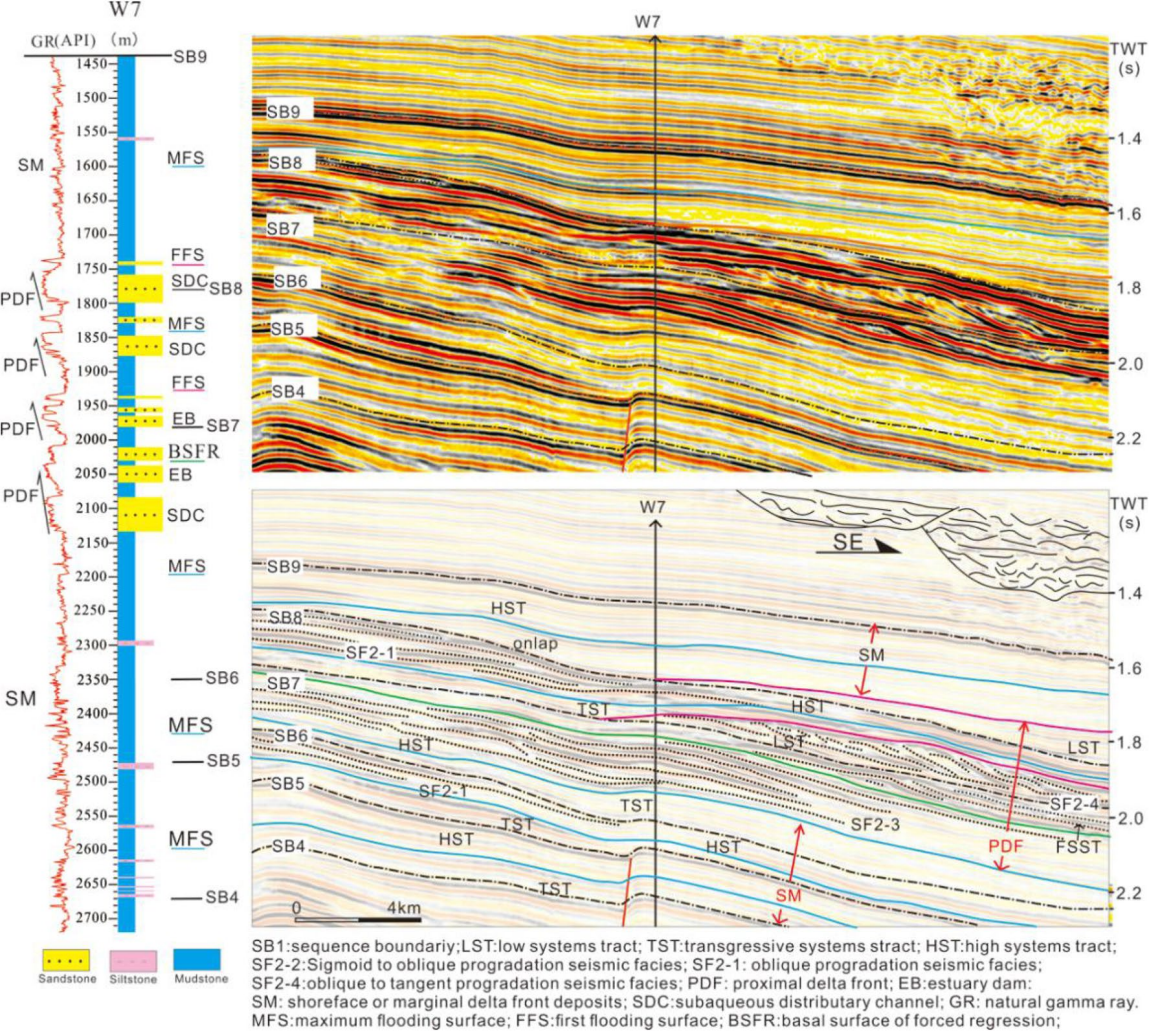
An interpreted seismic profile calibrated to borehole W7 demonstrating the deposits of the meandering river delta front from S4-S8 in the northwest of Baiyun Sag (see Fig. 1b for location of the profile). The proximal delta front deposits were located at the Highstand and Falling-stage systems tract of S6, the entire S7 and the Lowstand of S8, and exhibit characteristic thick bedded forests with upward-coarsening sand bodies (borehole W7 log curve).

Large-scale progradation reflections can be observed on the seismic profile of the Baiyun Sag in the Highstand and Falling-stage systems tract of S6 (Fig. 9), while thick box or funnel-shaped sandstone is identified for the well logs (Figs. 5 and 9). This indicates the subaqueous distributary channel and estuary dam deposits, which is distinct to that of the predelta to shallow shelf deposits in S3–S5. The thick-bedded sandstone indicates the abundant supply of the source and a high deposition rate. Studies have determined a rate of deposition of approximately 5 cm/ky^47^ at the end of S6. Also at this time, the global climate changed from a relatively warm to a colder stage, resulting in a large-scale decline in sea level in the Pearl River Mouth Basin and a large amount of clastics prograding to the Baiyun Sag, thus forming the thick delta front deposits.

Following the large-scale regression of S6, the sea level began to rise in S7, and the delta front retreated to the southern region of the Panyu Low Uplift. However, thick wedge-shaped progradation reflections are evident on the seismic profile of the northwest of Baiyun Sag in the Lowstand systems tract of S7, corresponding to estuary dam deposits in the well logs (Figs. 5 and 9). This may be attributed to the gradual advancement of the transgression from the east to west of the study area in S7 as well as the gradual evolvement of the northwest of Baiyun Sag into predelta to shallow shelf deposits. During the deposition of S8, the delta front retreated to the northern zone of the Panyu Low Uplift, corresponding to the strengthening of the transgression. There were also multiple incised channels perpendicular to the shoreline at the deep-water continental slope in the northwest of the Baiyun Sag. The root-mean-square amplitude slices do not clearly reveal the flow direction of the subaqueous distributary channel, while the delta was observed to extend from the northeast to southwest (Fig. 8b). This may be related to the influence of the waves. In particular, the Panyu Low Uplift is a high point on the topography, and it is greatly affected by the waves, resulting in a lobate delta on the plane.

## Discussion

The sequence structure and sedimentary evolution of the delta are restricted by several factors, including sea level change, sediment supply, tectonic movement and paleoclimate^48–50^. In order to reveal the evolution and formation mechanism of the delta sedimentary system in the study area, it is necessary to perform a comprehensive analysis of the above control factors.

### Sea level change

**Figure 10:**
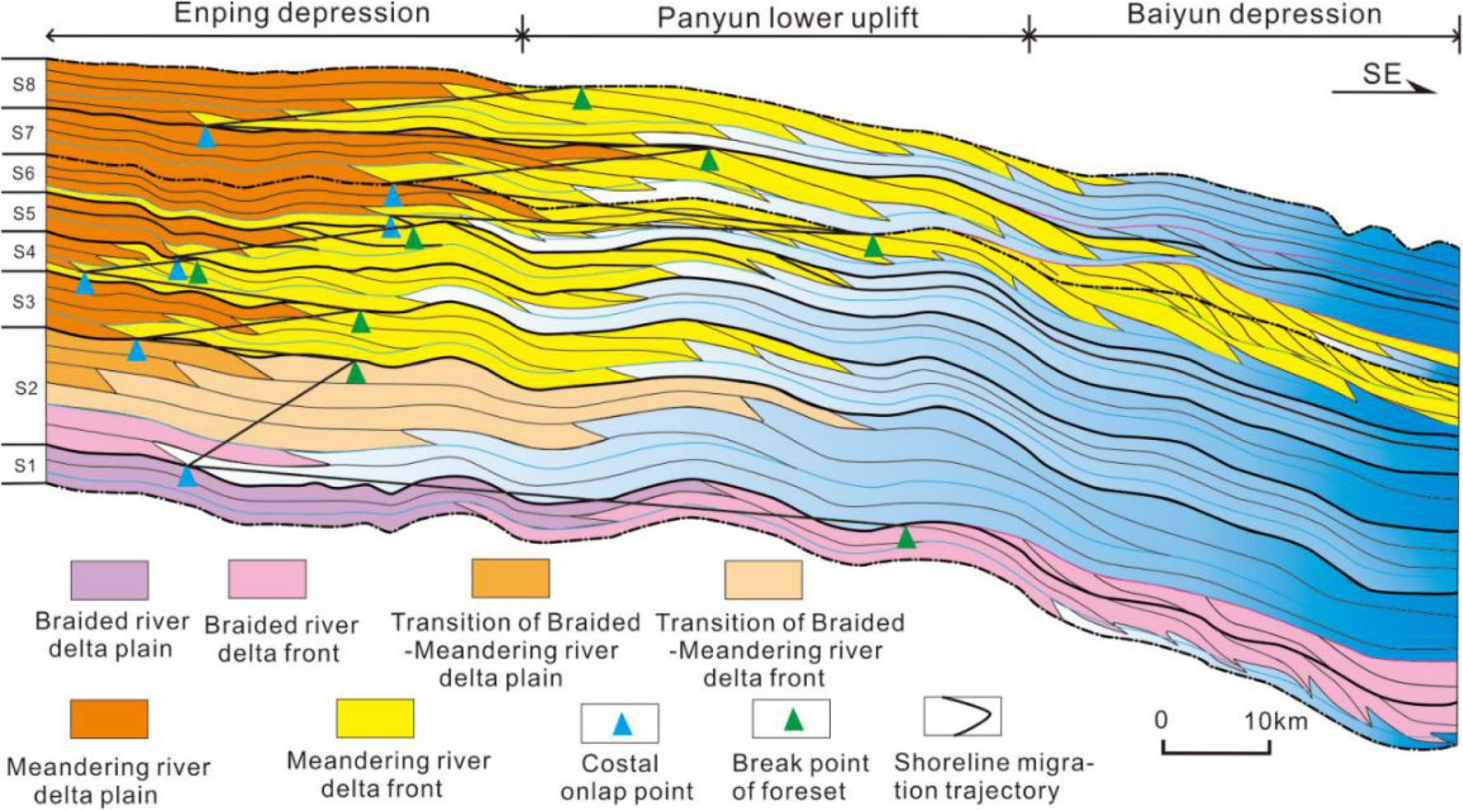
Depositional and evolution model of the braided and meandering river deltas in the Early-Mid Miocene on the northern Pearl River Mouth basin, based on the identification of deltaic deposits in the wells and cores. Tracking the costal onlap points and break points of the foreset on 3D seismic profiles can establish the shoreline migration trajectory, which can be compared to global sea level changes^51,52^.

**Figure 11:**
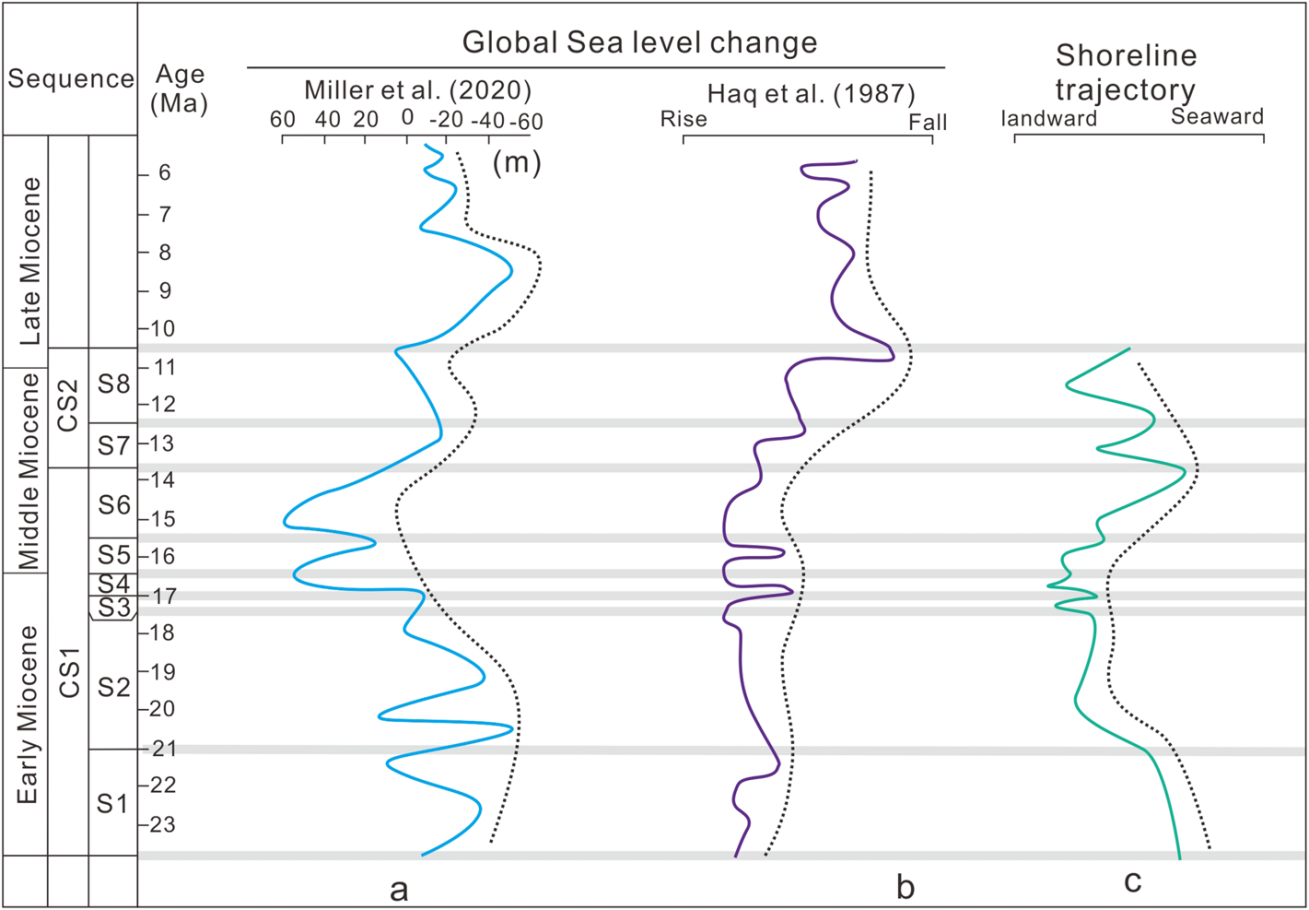
The comparison of shoreline trajectory and global sea level change in the Early-Mid Miocene on the northern Pearl River Mouth basin. Two typical global sea level curves^51,52^ are listed in the figure and they are partly related to the changes of shoreline trajectory in the study area. The gray lines were the sequence boundary of each sequence.

Based on the previous analysis of the sedimentary systems of the sequences and systems tract, a depositional model of the study area was established. By tracing the costal onlap points and slope break points of the different sequences and sedimentary system, a shoreline migration trajectory was plotted, which can reflect the relative sea level changes in the northern Pearl River Mouth Basin (Figs. 10 and 11). Both of the global sea level change curves from Haq et al.^51^ and Miller et al.^52^ are used as references to compare with the shoreline migration trajectory of the study area.

In the early Miocene (23.8 Ma), the shoreline was located in the southern region of the Panyu Low Uplift, while the delta front was located at the northwest of Baiyun Sag (Fig. 10). This indicates that the sea level of the study area was extremely low, however, both of the global sea level changes showed the lowest points in 21 Ma^52^ (Fig. 11a) and 22 Ma^51^ (Fig. 11b). The possible reason was that the migration of shoreline was not influenced by the global sea level change but by the tectonic movement of the rifting ridge transition from the north to south of the South China Sea. At the beginning of S2, the shoreline suddenly jumped to the north of the study area, and the delta front also retreated to the southern of Enping Sag (Fig. 10), reflecting rapid transgression. This is consistent with the rising global sea level (Fig. 11b) and is different from the Miller et al.^52^ sea level change, which fluctuated sharply in this stage (Fig. 11a). This indicated that the retrogradation of deltas in S2 was the results of global sea level rise^53^. During the deposition of S3–S5, the shoreline migrated slightly in the Enping depression, reflecting sea level eustacy modestly. This is also consistent with the total trend of global sea level (Fig. 11a,b), but each change in sequence is different from that. In the early Middle Miocene (S6), the shoreline migrated to the south of the study area, and the delta front prograded to the southern region of the Panyu Low Uplift. In the Falling-stage systems tract of S6, the thick delta front deposited in the northwest of the Baiyun Sag suggested the large-scale regression in the study area, coinciding with the declining global sea level (Fig. 11a,b). Previous studies suggested that the global sea level had dropped by about 53–69 m during 16.5–13.9 Ma^54^, while the average sea level of the Pearl River Mouth Basin dropped by nearly 100 m^55^, resulting in the migration of the shoreline to the shelf edge (Fig. 10). Massive clastics were transported to the shelf edge, forming a thick shelf edge delta^56^. This regression may be related to the formation of the Arctic and Antarctic ice ages^56,57^, and at least 90% of the East Antarctic Icesheets was formed during the middle Miocene^54^. During the deposition of S7–S8, the shoreline migrated to the north of the study area, and the delta front retreated to the southern zone of the Panyu Low Uplift (Fig. 10). This implies a second transgression with a smaller scale than the previous (in S2), and it is consistent with total trend of the Miller et al.^52^ sea level change, but is different from that of the Haq et al.^51^.

In conclusion, in the early stage of CS1 (S1), the delta migration is in conflict with both of the global sea level changes. In the middle and late CSI stages (S2–S6), the shoreline migration and depositional evolution of the deltas are mostly controlled by the eustatic cycles of the global sea level. During the deposition of CS2, the Miller et al.^52^ sea level change curve could explain the changes of shoreline migration in the study area.

### Tectonic subsidence and sediment supply

From the Late Oligocene to the Early Miocene (S1), large-scale tectonic movement occurred in the South China Sea, and the deep mantle uplift caused strong thermal attenuation and subsidence. At the same time, the rifting ridge transitioned from the north to south of the South China Sea, while the shelf break jumped from the south to the north of the Baiyun Sag. The rapid subsidence of the Pearl River Mouth basin (Fig. 11) led to large-scale transgression following the deposition of S1^27^, and the shallow sea shelf to deep water slope deposits covered the study area^42^. This consequently resulted in the formation of a structural foundation for the development of deltas in the continental shelf. After the deposition of S7, the subsidence rate of the basin weakened and gradually stabilized. The shelf break was located at the southern of the Panyu Low Uplift, and there was a large area of river-delta deposits in the northern shelf.

In the Early-Mid Miocene, the Paleo-pearl River was the main source provider of the Pearl River Mouth Basin, and its development affected the property and sedimentary filling characteristics of the basin^42,58^. During the deposition of S1–S2 (LST–TST), the braided river delta was dominated by gravelly coarse sandstone, with an average net-gross ratio of 0.61 and an average sandstone thickness of 79.4 m, indicating that the clastics are from the near-source accumulation. At the beginning of the Early Miocene, clastics of the sedimentary rocks and metamorphic rocks of South China were carried by the Paleo-pearl River to accumulate in the basin^59^. Evidence from benthic foraminifera δ^18^O, the black carbon δ^13^C and chemical index of alteration (CIA) in the sediments of ODP1148 station indicates that the chemical weathering intensity in the source region was high and the climate was warm and humid in the early Miocene, which were conducive to the weathering and denudation of the parent rock^59–63^.

During the Highland systems tract of S2, the average of net-gross ratio and sandstone thickness of the delta were significantly reduced to 0.23 and 45.4 m, respectively. The delta retreated to the north and became a braided-meandering river transitional delta. With the large-scale transgression, the Paleo-pearl river expanded westward, increasing the clastics from the intermediate-acid volcanics and granite of the Yanshan fold belt along the South China coast^64^. The humidity decreased from the early Miocene^63^ and the temperature dropped by about 0.7–1.2 °C since the middle Miocene^52^. During the deposition of S3–S6, the average of net-gross ratio and sandstone thickness were 0.26 and 49.41 m, indicating the distal source of the clastics. During the Middle Miocene, the Paleo-pearl River could extend to the Tibet Plateau and the Yunnan-Guizhou Plateau^64–66^, and the Guijiang, Liujiang and Beipan Rivers flowed into the Paleo-pearl River^67^, providing a material basis for the formation of the delta.

During the deposition of CS2, the sedimentation rate of the Pearl River Mouth Basin weakened, and the paleoclimate gradually transformed from warm and humid in the Early Miocene to dry and cold^68^, which eased the weathering of the parent rock in the provenance area and reduced the sediment supply. Therefore, the delta in the study area gradually degraded towards the continent, and the shoreline migrated to the land. The rapid rise in the sea level enhanced the wave action in the study area, which laid the foundation for the formation of a wave-controlled meandering river delta systems.

## Conclusion

1. Fluvial and delta depositional systems were widely developed in the Early-Mid Miocene across the northern shelf of the Pearl River Mouth Basin. Multiple transgression-regression sedimentary cycles were formed with the sea level change. Two composite (CS1–CS2) and eight (S1–S8) sequences are classified in the Early-Mid Miocene succession. The Lowstand-Transgressive systems tract of S1–S2 were the braided river delta deposits and the Highstand systems tract of S2 was a transition delta deposits of braided and meandering river, the deposition of S3–S8 was the meandering river delta deposits.
2. Two types of deltaic systems are recognized in the succession. Braided river delta systems are characterized by medium to thick stacked pebbly coarse–fine and poorly sorted and rounded sandstones of distributary channel and deltaic front deposits, whereas meandering river delta systems are characterized by thin delta plain and thick delta front deposits composed of medium to thinly pebbly well sorted and rounded medium to fine sandstones. Braided river deltaic deposits mainly developed in S1 and the Lowstand and Transgressive systems tract of S2, while the transitional type of the braided and meandering river deltas formed in the Transgressive and Highstand systems tracts of S2, and the meandering river delta systems dominated S3–S8.
3. The transition of the braided river delta to the meandering river delta and backward or forward migration of the delta systems in the study area were constrained by interplay of sea level change, tectonic subsidence and sediment supply. During the deposition of S1–S2, the braided river delta systems were mainly controlled by tectonic subsidence and sedimentary provenance. The sediments provided by the Paleo-pearl river were deposited in the Baiyunxi Sag, forming large-scale braided river delta systems. During the deposition of S3–S6, the development of the meandering river delta systems was related to the global sea level rise. During the transgression, the deltas retreated to the Enping Sag; and then progressed to the northwest of the Baiyun Sag in the regression period. During the deposition of S7–S8, the rise of global sea level and wave effects have a significant influence on the development of the wave-controlled deltaic systems.
